# Perinatal Outcome in Women with Hypertensive Disorders of Pregnancy: A Retrospective Cohort Study

**DOI:** 10.1155/2015/208043

**Published:** 2015-01-08

**Authors:** Gezehagn Endeshaw, Yifru Berhan

**Affiliations:** ^1^Hawassa University, 1560 Hawassa, Ethiopia; ^2^Hawassa University College of Medicine and Health Sciences, 1560 Hawassa, Ethiopia

## Abstract

*Background*. Hypertensive disorders of pregnancy (HDP) are multisystem diseases known to increase the risk of perinatal mortality worldwide, with a significant proportion of these deaths occurring in low income countries. However, little is known about the obstetric and treatment predictors of perinatal mortality in women with HDP. *Methods*. A retrospective cohort study design was used to include 1015 hypertensive pregnant women who gave birth to 1110 babies between 2008 and 2013 in three university teaching hospitals. Bivariate and multivariate regression models were used to estimate the associations between selected predictor variables and perinatal mortality taking the onset of HDP illness to death or discharge from the hospital as the time period. *Results*. There were 322 perinatal deaths resulting in a perinatal mortality rate (PMR) of 290/1000 total births. The proportion of stillbirths was more than 4-fold higher than early neonatal deaths (81% versus 19%). The multivariate analysis demonstrated that multiparity (OR, 1.6; 95% CI, 1.12–228), grand multiparity (OR, 2.8; 95% CI, 1.55–4.92), preterm (OR, 1.5; 95% CI, 1.02–2.35) and very preterm gestational age (OR, 7.7; 95% CI, 5.26–11.20), lack of antenatal care (OR, 2.0; 95% CI, 1.43–2.67), having eclampsia (OR, 4.1; 95% CI, 2.85–6.04), antepartum or before (OR, 6.6; 95% CI, 3.40–12.75) and intrapartum onset of HDP (OR, 4.0; 95% CI, 1.99–8.04), raised SGOT level (OR, 2.3; 95% CI, 1.30–3.91), vaginal delivery (OR, 5.3; 95% CI, 2.93–9.54), low fetal birth weight (OR, 4.3; 95% CI, 2.56–7.23), and maternal death (OR, 12.8; 95% CI, 2.99–54.49) were independent predictors of perinatal mortality. *Conclusion*. This study showed that the PMR of HDP was among the highest in the world. Parity, gestational age, type and onset of HDP, mode of delivery, birthweight, and maternal outcome were strong predictors of perinatal mortality.

## 1. Introduction

Hypertensive disorders of pregnancy (HDP) are multisystem diseases, which include chronic (preexisting) hypertension, gestational hypertension, preeclampsia, eclampsia, and preeclampsia superimposed on chronic hypertension [1]. These disorders may complicate 5%–10% of all pregnancies [2] and are leading causes of maternal and perinatal mortality and morbidity worldwide [3].

The high perinatal mortality in women with HDP is mainly due to premature delivery and growth restriction [4, 5]. A secondary analysis from the World Health Organization (WHO) multicountry survey has shown that there were about 3- and 5-fold increased risk of perinatal death in women with preeclampsia and eclampsia, respectively, as compared to women with no preeclampsia/eclampsia [3]. Specifically, the perinatal mortality in women with hypertensive disorders was reported as 230/1000 births from Pakistan [6], 144/1000 births from Turkey [7], 165/1000 births from Addis Ababa [8], and 317/1000 births from Jimma/Ethiopia [9]. Another study, which included only eclamptic mothers, also showed the high perinatal mortality [10].

Although there is a large body of literature that described the magnitude and associated complications of HDP, little is done to assess the predictors of perinatal mortality, particularly in low and middle income countries [11–13]. This is despite the fact that the majority of perinatal deaths due to complications of HDP have occurred in the low and middle income countries [6–10, 14]. Similarly, the authors of this study could not find a published study on HDP in the Southern Regional State of Ethiopia. Furthermore, the authors observed high perinatal mortality in the hospitals where they have been working. This study was planned with an objective of determining the predictors of perinatal mortality among women with HDP.

## 2. Methods

### 2.1. Study Setting and Design

This analysis was done using data from three university teaching hospitals in the Southern Regional State of Ethiopia (Hawassa Referral Hospital, Hosanna Hospital, and Yirgalem Hospital) from 2008 to 2013. During this period, a total of 30,750 babies were delivered after 28 weeks of gestational age, of which 1098 women were diagnosed to have HDP. A retrospective cohort study design was used to include the patient's data from the onset of HDP to the time end of treatment was declared (mother discharged as a cure or for death). This study included all eligible women with HDP admitted to the study hospitals during the study period. The exclusion criteria were baby delivery before 28 weeks of gestation, lost or incomplete data, or mother death on arrival.

### 2.2. Variables and Data Collection

An association of perinatal mortality was assessed for maternal age, parity, gestational age, antenatal care, number of fetuses, type of HDP, onset of HDP, severity symptoms of HDP, proteinuria, hemoglobin, platelet count, creatinine, serum oxaloacetic transaminase (SGOT) level, labor onset, type of anticonvulsant or antihypertensive given, mode of delivery, fetal birth weight, and maternal outcome.

For each study hospital, nine nurse data collectors were recruited and trained. To access the detailed data in the patient chart, the delivery log book was used as an entry point to identify the HDP patients by their card number. A structured data collecting format was prepared and used to abstract relevant data from the included patients' charts starting from the onset of signs and symptoms of HDP to the time end date was declared.

### 2.3. Data Processing and Analysis

After completeness was checked, data were coded, entered, and analyzed using computer data analysis software program (SPSS version 20). Bivariate and multivariate regression models were used to estimate the associations between selected predictor variables and perinatal mortality. A statistically significant association was considered when the odds ratio (OR) 95% confidence interval did not include the number 1. Variables which did not show statistical significance in the univariate analysis were excluded from the multivariate analysis.

### 2.4. Operational Definitions

The finding of raising blood pressure or severe symptoms of hypertension with significant proteinuria was enough to diagnose preeclampsia. The occurrence of convulsion or coma not attributable to other causes defined eclampsia. Chronic hypertension was diagnosed when the women were found to have hypertension before the occurrence of pregnancy or before 20 weeks of gestation. A new onset or worsening of proteinuria and/or worsening of hypertension in women with a known chronic hypertension defined superimposed preeclampsia. The development of an elevated blood pressure during the second half of pregnancy without proteinuria and without severity symptoms was used to diagnose gestational hypertension [1, 3, 5].

In this study, other HDP included all women with chronic hypertension, gestational hypertension, and superimposed preeclampsia. Pregnant or postpartum women with a systolic blood pressure (BP) < 140 mmHg and diastolic BP < 90 mmHg were categorized as normotensive. A systolic BP ≥ 140 mmHg and diastolic BP ≥ 90 mmHg measured twice six hours apart defined mild to moderate hypertension. A single record of systolic BP ≥ 160 mmHg and diastolic BP ≥ 110 mmHg was enough to define severe hypertension. Headache, blurred vision, epigastric pain, and vomiting were taken as severity symptoms of HDP.

Significant proteinuria was considered when the qualitative test revealed +2 and above or +1 if the specific gravity was <1.020. Creatinine level <1 and 1+ mg/dL and SGOT level raised by < or >2-fold from the baseline were included in the analysis as a proxy indicator for status of renal function and liver function, respectively. The normal value of SGOT level was <36 IU/L in all study hospitals. Platelet count was also dichotomized as < or >100,000/mm^3^. Degree of anemia was categorized as severe to moderate (<10 gm/dL), mild (10–11.9 gm/dL), and no anemia (12+ gm/dL).

All fetal deaths in utero (stillbirth) and all early neonatal deaths (deaths that occurred in the first week of neonatal life before discharge from the study hospital) after 28 weeks of gestation were included in the perinatal mortality. The perinatal mortality rate (PMR) was determined out of 1000 total births among women included in this study.

### 2.5. Ethical Consideration

Ethical clearance was obtained from institutional review board of Hawassa University College of Medicine and Health Sciences. Since the study was retrospective by design, written consent from patients was not required. Anonymity was secured by analyzing and presenting the data in aggregate.

## 3. Results

This study included 1015 hypertensive pregnant women who gave birth to 1110 babies (95 sets of twins) after 28 weeks of gestational age. The detailed description is presented in a separate analysis. Pertinent to this analysis, there were 322 perinatal deaths resulting in a perinatal mortality rate of 290/1000 total births. The proportion of stillbirths was more than 4-fold higher than early neonatal deaths: 261 (81%) stillbirths versus 61 (18.9%) early neonatal deaths. Twin accounted for 46 (14.3%) of the total perinatal deaths.

The distribution of perinatal mortality by type of HDP was preeclampsia 159 (49.4%), eclampsia 143 (44.4), and other types of HDP 20 (6.2%). However, the case fatality rate of preeclampsia (24%) was significantly lower than eclampsia (38.2%) and other types of HDP (33.3%). The majority of perinatal deaths (261/322, 81%) occurred among women with antepartum onset of HDP. Nearly two-thirds of the perinatal deaths were either very preterm or preterm (201/322, 62%). Among the total perinatal deaths in antepartum period, 71% (184/261) were either very preterm or preterm. In intrapartum and antepartum onset of HDP, the proportion of perinatal deaths before term was about 39%. The premature perinatal deaths were more pronounced in preeclamptic than in eclamptic women (31% versus 61%).

The median gestational ages for perinatal deaths and survivors in women with preeclampsia were 32 and 38 weeks, respectively. In eclamptic women, however, the median gestational ages for perinatal deaths and survivors were term (37 versus 38 weeks). Similarly, the median birth weight of perinatal deaths of preeclamptic mothers was nearly 2-fold lower than the survivors (1.5 kg versus 2.7 kg). Among eclamptic women, the median birth weight of perinatal deaths was relatively close to the survivors (2.3 versus 2.7 kg). Overall, nearly three-fourths of babies who died in the perinatal period were having birth weight below 2.5 kg (233/322, 72%). In the majority of perinatal deaths, their mothers' hemoglobin level (197/322, 61%), platelet count (216/322, 67%), and SGOT level (169/322, 52%) were in the normal range.

Table 1 shows the logistic regression of the selected variables as potential determinants for perinatal mortality. In the bivariate analysis, perinatal mortality was found to be weakly associated with multiparty (crude OR, 1.4; 95% CI, 1.04–1.80) and strongly associated with grand multiparity (COR, 2.3; 95% CI, 1.44–3.68). Other variables, which have shown strong association with perinatal mortality, were very preterm delivery (COR, 8.1; 95% CI, 5.82–11.22), lack of antenatal care follow-up (COR, 2.3; 95% CI, 1.75–2.97), having eclampsia (COR, 2.0; 95% CI, 1.52–2.63), antepartum onset of HDP (COR, 2.6; 95% CI, 1.57–4.38), and the highest diastolic blood pressure (BP) being <90 mmHg (COR, 2.1; 95% CI, 1.11–4.00). There was also a statistically significant association of perinatal mortality with the highest systolic BP ≥ 160 mmHg (COR, 1.5; 95% CI, 1.07–1.99) and highest diastolic BP ≥ 110 mmHg (COR, 1.7; 95% CI, 1.33–2.31).

In the multivariate analysis, the perinatal mortality has increased by about 1.6- and 2.8-fold among multiparous and grand multiparous women, respectively. The risk of perinatal mortality in very preterm babies was 7.7-fold higher than term babies. Lack of antenatal care and having eclampsia increased the risk of perinatal mortality by more than 2- and 4-fold. Antepartum and intrapartum onset of HDP also independently predicted the chance of perinatal mortality by 6.6- and 4-fold as compared to postpartum onset of HDP.

In Table 2, the association of perinatal mortality with selected laboratory findings, treatment modalities, and the outcome is presented. Among selected laboratory tests, the bivariate analysis has demonstrated significant association of perinatal mortality with the lowest platelet count of <100,000/mm^3^ (COR, 2.3; 95% CI, 1.66–3.31), highest creatinine level of ≥1 mg/dL (COR, 1.5; 95% CI, 1.05–2.03), and highest SGOT raised by ≥2-fold from the normal level (COR, 2.9; 95% CI, 1.96–4.22).

Perinatal mortality was higher among women whose labor was either spontaneously initiated (COR, 2.3; 95% CI, 1.49–3.78) or induced (COR, 4.9; 95% CI, 3.16–7.49). There was also a strong association of perinatal mortality with vaginal delivery (COR, 4.5; 95% CI, 3.32–6.13). Birth weight < 2.5 kg was associated with four times increased risk of mortality during the perinatal period (COR, 4.1; 95% CI, 3.02–5.45). The odds of perinatal mortality among mothers who lost their life were more than 7-fold higher than alive mothers (COR, 7.1; 95% CI, 3.79–13.38). In the multivariate analysis, however, a strong association of perinatal mortality with highest SGOT raised by ≥2-fold (OR, 2.3), vaginal delivery (OR, 5.3), low birth weight (OR, 4.3), and maternal death (OR, 12.8) was demonstrated.

As shown in Table 3, a multivariate analysis was done by disaggregating the perinatal mortality into stillbirth and early neonatal mortality (ENM). The independent predictors of stillbirth include multiparity (OR, 1.6), grand multiparity (OR, 2.6), very preterm gestational age (OR, 6.5), lack of antenatal care (OR, 2.1), developing eclampsia (OR, 4.1), and antepartum (OR, 7.8) or intrapartum (OR, 5.0) onset of HDP. Among the laboratory findings, lowest platelet count < 100,000/mm^3^ (OR, 2.2) and SGOT level raised by ≥2-fold (OR, 2.2) were independently associated with stillbirths. There was also a strong association of stillbirth with vaginal delivery (OR, 7.1), birth weight < 2.5 kg (OR, 4.0), and maternal death (OR, 10.4).

On the other hand, the independent predictors of ENM were very preterm gestational age (OR, 3.6), having eclampsia (OR, 2.9), vaginal delivery (OR, 4.1), birth weight < 2.5 kg (OR, 6.2), and maternal death (OR, 11.7). Otherwise, the statistically significant association of perinatal mortality, stillbirth, and ENM was not observed with maternal age, number of fetuses, HDP severity symptoms, significant proteinuria, hemoglobin level, and type of anticonvulsant or antihypertensive given.

## 4. Discussion

Consistent with previous studies' findings [3, 7, 15–17], this study has shown a high perinatal mortality among women with HDP. However, the PMR in this study (290/1000 births) was lower than the report from Jimma/Ethiopia [9] but higher than the reports from Pakistan [6], Turkey [7], and Addis Ababa [8].

Specific to the objective of this study, the analysis demonstrated that the independent predictor variables of perinatal mortality were parity, gestational age, antenatal care, type and onset of HDP, SGOT level, mode of delivery, fetal birth weight, and maternal outcome. An increase in the risk of perinatal mortality with an increase in parity was observed in this study. A separate analysis has also shown the increase in maternal mortality with an increase in parity [18]. This is despite the fact that HDP commonly occur among primigravida women [15, 19].

The significantly increased risk of perinatal mortality among babies with low gestational age and low birth weight in this study is consistent with several other studies [4, 5, 20, 21]. The finding of a strong association of perinatal mortality with low gestational age and low birth weight is probably in line with the increased risk of perinatal mortality among babies with antepartum onset of HDP. It was observed that nearly three-fourths of the perinatal deaths (71%) in women with antepartum onset of HDP were either preterm or very preterm at birth. The implication is that HDP has probably exposed several babies to premature delivery and its complications. Previous study also reported the increased risk of premature delivery in women with HDP [22]. Furthermore, other studies focusing only on eclamptic women reported that perinatal deaths were caused by prematurity in 68% [20] and 43% [21].

On the other hand, significantly increased odds of perinatal mortality were observed among women with eclampsia, which was consistent with other studies [15, 23]. This is probably because of the severe nature of the eclampsia disease, which usually complicates by severe intrauterine asphyxia, severe placental abruption, and neonatal sepsis [24]. However, the proportion of premature perinatal mortality was about 2-fold higher in women with preeclampsia than women with eclampsia. This finding might be due to the fact that in the majority of the cases preeclampsia is the initial disease before eclampsia, which may be the reason to come to the hospital earlier than women with eclampsia and get the pregnancy terminated. This study has also shown that preterm delivery rate was nearly twice higher in preeclamptic than in eclamptic women.

This study has also shown the independent association of perinatal mortality with lack of antenatal care, which is probably an indirect evidence of delay in recognition and intervention in the affected mothers. This is because the delay in diagnosis and delay in providing treatment in the early stage of the disease are likely to progress to severe stage of the disease like eclampsia, which was in turn found to have a strong association with perinatal mortality. Previous studies also reported reduction in perinatal mortality among hypertensive women who had antenatal care [25, 26].

Among the selected laboratory tests to assess the severity of HDP, only SGOT level ≥2-fold raise from the normal range was an independent predictor of perinatal mortality. The platelet count and creatinine levels were predictors of perinatal mortality in the unadjusted analysis, but hemoglobin was not. This is probably because the intrauterine wellbeing of the fetus is mainly dependent on the adequacy of uteroplacental blood flow and placental function [27], which may not be much influenced by the maternal renal and hematologic function derangements. Previous study, however, reported an increase in the risk of perinatal mortality in women with proteinuria [28]. The implication is that the association of maternal organs function derangements due to HDP needs further investigation.

Similarly, this study did not show statistically significant association of perinatal mortality with a type of anticonvulsant in both bivariate and multivariate analyses. One previous study also reported that there was no difference in perinatal mortality between the diazepam and magnesium sulphate groups [29]. Another study, however, reported nearly 3-fold reduction in perinatal mortality among magnesium sulphate group [30]. It was also noted that magnesium sulphate is superior to diazepam in preventing and controlling convulsion in women with HDP [31–33], which is indirectly preventing the most perinatal deadly complication (eclampsia). Nevertheless, the inconsistent findings of the effect of magnesium sulphate on perinatal mortality invoke meta-analysis.

The strong association of perinatal mortality with vaginal delivery may not necessarily show the increased risk of perinatal mortality among babies delivered via the vaginal route. This is because 80% of the stillbirths occurred before the onset of labor or before caesarean delivery was considered. Otherwise, caesarean delivery is known to result in expeditious delivery and probably has reduced the risk HDP related complications and perinatal mortality.

The very strong association of perinatal mortality with maternal mortality may strengthen the finding of significant association of perinatal mortality with eclampsia, which was an identified risk factor for both perinatal and maternal deaths as this study and previous studies showed [18, 20, 21, 34–36].

This study has several limitations. Since the study was retrospective by design, we could not get some data on social and service related factors. In other words, the quality of neonatal care was not assessed, which has probably contributed to high neonatal mortality among preterm and/or asphyxiated babies. Similarly, due to lack of documented data, the measurements for antepartum and postpartum care were not comprehensive, particularly in relation to referral and medications unavailability.

In conclusion, this study showed that the PMR of HDP was among the highest in the world. The multivariate analysis demonstrated that high parity, low gestational age, lack of antenatal care, having eclampsia, predelivery onset of HDP, raised SGOT level, vaginal delivery, low fetal birth weight, and maternal death were independent predictors of perinatal mortality. The majority of perinatal mortality predictors were also predictors of stillbirths. Since the strong association of perinatal mortality with eclampsia (a late complication of HDP in the majority) [37, 38] and lack of antenatal care is an indirect evidence for the delay in utilization of obstetric services, pregnant women in the study area need to be educated on the importance of antenatal care and HDP symptoms.

## Figures and Tables

**Table 1:** Predictors of perinatal mortality among women with hypertensive disorders of pregnancy (HDP), Ethiopia, 2008–2013.

Variables^*^	Total	Perinatal deaths (%)	Crude OR (95% CI)	Adjusted OR (95% CI)
Maternal age (years)				
15–19	57	31.6	1.0 (0.59–1.85)	
20–34	876	31.3	1	
35–49	82	36.6	1.2 (0.79–1.98)	
Parity				
Primigravida	536	27.4	1	1
Multipara (I–IV)	400	34.5	1.4 (1.04–1.80)^¥^	1.6 (1.12–2.228)^‡^
Grand multipara (V+)	79	46.8	2.3 (1.44–3.68)^‡^	2.8 (1.55–4.92)^‡^
Gestational age (weeks)				
Very preterm (<34)	240	63.8	8.1 (5.82–11.22)^†^	7.7 (5.26–11.20)^†^
Preterm (34–36)	193	24.9	1.5 (1.04–2.23)^¥^	1.5 (1.02–2.35)^¥^
Term + (≥37)	677	17.9	1	1
Number of fetuses				
Singleton	920	30.0	1	
Twin	190	24.2	0.7 (0.52–1.07)	
Antenatal care				
Yes	633	22.7	1	1
No	382	40.0	2.3 (1.75–2.97)^†^	2.0 (1.43–2.67)^†^
Type of HDP				
Preeclampsia	612	26.0	1	1
Eclampsia	346	41.3	2.0 (1.52–2.63)^†^	4.1 (2.85–6.04)^†^
Other HDP	57	35.1	1.6 (0.92–2.86)	1.3 (0.65–2.49)
Onset of HDP				
Antepartum or before	721	36.2	2.6 (1.57–4.38)^†^	6.6 (3.40–12.75)^†^
Intrapartum	183	22.9	1.4 (0.77–2.55)	4.0 (1.99–8.04)^†^
Postpartum	111	17.1	1	1
HDP severity symptoms				
Yes (one or more)	847	32.8	1.3 (0.92–1.92)	
None	168	26.2	1	
Highest systolic BP				
<140 mmHg	60	36.7	1.5 (0.86–2.70)	1.0 (0.44–2.24)
140–159	270	25.9	1	1
160+	685	33.6	1.5 (1.07–1.99)^¥^	1.0 (0.67–1.55)
Highest diastolic BP				
<90 mmHg	39	43.6	2.1 (1.11–4.00)^¥^	2.3 (0.88–5.79)
90–109	425	24.5	1	1
110+	551	36.5	1.7 (1.33–2.31)^†^	1.4 (0.96–2.03)

^*¥*^ *P* < 0.05; ^‡^ *P* ≤ 0.001; ^†^ *P* < 0.0001. BP: blood pressure. ^*^All variables describe the mother's condition except gestational age and fetuses number. ^**^Adjusted for parity, gestational age, antenatal care, type of HDP, onset of HDP, highest systolic BP, and highest diastolic BP.

**Table 2:** Association of perinatal mortality with selected laboratory findings, treatment modalities, and outcome among women with hypertensive disorders of pregnancy, Ethiopia, 2008–2013.

Variables^*^	Total	Perinatal deaths (%)	Crude HR (95% CI)	Adjusted^**^ HR (95% CI)
Hemoglobin level (gm/dL)				
<10.0	158	36.1	1.0 (0.71–1.51)	
10–11.9	255	26.7	0.7 (0.51–1.00)	
≥12	602	32.7	1	
Platelet count in mm3				
<100	223	47.5	2.3 (1.66–3.31)^†^	1.5 (0.83–2.85)
≥100	792	27.3	1	1
Proteinuria (qualitative)				
Insignificant	266	27.1	1	
Significant	749	30.4	1.2 (0.89–1.68)	
Highest creatinine (gm/dL)				
<1.0	494	26.3	1	1
1.0+	521	36.8	1.5 (1.05–2.03)^¥^	0.8 (0.51–1.39)
Highest SGOT (IU/microl)				
<2-fold raise	689	24.5	1	1
≥2-fold raise	326	46.9	2.9 (1.96–4.22)^†^	2.3 (1.30–3.91)^‡^
Antihypertensive drug given				
Yes	811	32.8	1	
No	204	27.5	0.8 (0.55–1.11)	
Anticonvulsant drug given				
MgSO_4_	641	30.6	1	
Diazepam	374	33.7	1.1 (0.81–1.41)	
Labor onset				
Spontaneous	331	26.3	2.3 (1.49–3.78)^†^	0.7 (0.25–2.07)
Induced	504	41.1	4.9 (3.16–7.49)^†^	1.6 (0.69–3.81)
Direct C/S	180	15.6	1	1
Mode of delivery				
Vaginal	625	41.1	4.5 (3.32–6.13)^†^	5.3 (2.93–9.54)^†^
Caesarean section	485	13.4	1	1
Birth weight (kg)				
<2.5	545	42.7	4.1 (3.02–5.45)^†^	4.3 (2.56–7.23)^†^
2.5+	565	15.7	1	1
Maternal outcome				
Alive on discharge	964	29.0	1	1
Dead	51	82.3	7.1 (3.79–13.38)^†^	12.8 (2.99–54.49)^‡^

^*¥*^ *P* < 0.05; ^‡^ *P* = 0.001; ^†^ *P* < 0.0001. ^*^All variables describe the mother's condition except mode of delivery and birth weight. ^**^Adjusted for platelet count, creatinine level, SGOT level, labor onset, mode of delivery, birth weight, and maternal outcome. C/S: Caesarean section. SGOT: serum glutamic oxaloacetic transaminase.

**Table 3:** Multivariate analysis of stillbirth and early neonatal mortality predictors in women with hypertensive disorders of pregnancy, Ethiopia, 2008–2013.

Variable	Stillbirths	Early neonatal deaths
	Adjusted OR (95% CI)	Adjusted OR (95% CI)
Parity		
Primipara	1	
Multipara	1.6 (1.09–2.33)^¥^	
Grand multipara	2.5 (1.34–4.71)^‡^	
Gestational age (weeks)		
Very preterm (<34)	6.5 (4.33–9.85)^†^	3.6 (1.49–8.76)^†^
Preterm (34–36)	1.4 (0.87–2.27)	0.7 (0.23–2.28)
Term + (≥37)	1	1
Antenatal care		
Yes	1	
No	2.1 (1.47–2.93)^†^	
Type of HDP		
Preeclampsia	1	1
Eclampsia	4.1 (2.69–6.09)^†^	2.9 (1.37–6.17)^‡^
Other HDP	1.5 (0.77–3.09)	0.8 (0.13–4.42)
Onset of HDP		
Antepartum or before	7.8 (3.59–16.86)^†^	
Intrapartum	5.0 (2.20–11.20)^†^	
Postpartum	1	
Lowest platelet count per mm3		
<100,000	1	
100,000+	2.2 (1.14–4.13)^¥^	
Highest SGOT (IU/microl)		
<2-fold raise	1	
≥2-fold raise	2.2 (1.21–3.89)^¥^	
Mode of delivery		
Vaginal	7.1 (3.48–14.39)^†^	4.1 (1.81–9.45)^†^
Caesarean section	1	1
Birth weight (kg)		
<2.5	4.0 (2.24–7.04)^†^	6.2 (2.31–16.57)^†^
2.5+	1	1
Maternal condition		
Alive on discharge	1	1
Dead	10.4 (2.15–50.83)^†^	11.7 (3.01–45.66)^†^

^*¥*^ *P* < 0.05; ^‡^ *P* = 0.001; ^†^ *P* < 0.0001.
